# The BOOST paediatric advance care planning intervention for adolescents with cancer and their parents: development, acceptability and feasibility

**DOI:** 10.1186/s12887-022-03247-9

**Published:** 2022-04-15

**Authors:** Anne van Driessche, Joni Gilissen, Aline De Vleminck, Marijke Kars, Jurrianne Fahner, Jutte van der Werff ten Bosch, Luc Deliens, Joachim Cohen, Kim Beernaert

**Affiliations:** 1 End-of-Life Care Research Group, Vrije Universiteit Brussel (VUB) & Ghent University, Laarbeeklaan 103, 1090 Brussels, Belgium; 2 Department of Family Medicine and Chronic Care, Vrije Universiteit Brussel (VUB), 1090 Brussels, Belgium; 3 Center of Expertise Palliative Care Utrecht, Julius Center of Health and Primary Care, UMC Utrecht, Universiteitsweg 100, 3584 CG Utrecht, the Netherlands; 4 Division of Paediatrics, Wilhelmina Children’s Hospital, UMC Utrecht, Utrecht, the Netherlands; 5 Department of Paediatric Hematology-Oncology, University Hospital Brussels, Vrije Universiteit Brussel (VUB), 1090 Jette, Belgium; 6 Department of Public Health and Primary Care, Ghent University, 9000 Ghent, Belgium

**Keywords:** Advance care planning, Paediatric palliative care, Paediatric oncology, Intervention development, Communication, Adolescent, Parent-adolescent communication

## Abstract

**Background:**

Although advance care planning (ACP) has been widely recommended to support patient and family engagement in understanding the patient’s values, preferences and goals of care, there are only a few models in paediatric oncology that capture ACP as a process of behaviour change. We aimed to develop and test the acceptability and feasibility of BOOST pACP (Benefits of Obtaining Ownership Systematically Together in paediatric Advance Care Planning) – an intervention to improve ACP in adolescents with cancer, their parents and paediatric oncologists.

**Methods:**

Several methods informed the intervention development process: 1) Problem identification**:** interviews with 11 healthcare professionals working in paediatric oncology; 2) Identification of evidence: literature review of existing pACP tools and barriers and facilitators in performing pACP; 3) Logic model and 4) Intervention design: collaborative expert meetings with researchers and professionals in pACP; 5a) Acceptability test of the materials: interviews with nine healthcare professionals, four adolescents and young adults with cancer and six parents; 5b) Feasibility test of core intervention components with three families, including interviews about their experiences.

**Results:**

The BOOST pACP intervention was iteratively developed and adapted, based on feedback from families, healthcare professionals, and pACP experts (e.g., components were changed, deleted, and added; formulation of themes and associated questions were amended to enhance acceptability). The core components of the BOOST pACP intervention include: four ACP conversation sessions with the adolescent and/or parent(s) provided by a trained facilitator, structured by interactive conversation cards covering different ACP themes, followed by a transfer of information from the intervention facilitator to the paediatric oncologist. Core intervention components were deemed feasible by all participating families.

**Conclusion:**

The BOOST pACP intervention was developed by close involvement of both adolescent patients and their parents, healthcare professionals and pACP experts. The final intervention and supporting materials are considered appropriate and feasible. Its effectiveness in improving parent-adolescent communication on ACP themes is currently being tested in a multi-centre randomised controlled trial. Researchers aiming to develop a complex psychosocial intervention for a vulnerable target group could use the step-by-step approach described in this paper.

**Supplementary Information:**

The online version contains supplementary material available at 10.1186/s12887-022-03247-9.

## Introduction

Many adolescents with cancer often require multiple medical procedures, hospitalisations or adjusted home care services with the potential to cure the illness and to maintain as normal a life as possible [1]. Families and clinicians must navigate serial uncertainties, from diagnosis through survivorship or end of life [2]. The interface between development, cancer, and treatment means that the impact is often persistent and can affect individual and family for many years [3]. With the complexity of these conditions, clinicians, parents and adolescents frequently face difficult decisions and conversations involving both current and future care and treatment options [4, 5]. Therefore, it is important to support family engagement in understanding the adolescent’s goals of care [5]. The lack of open communication between parents, adolescent and healthcare professionals about living with illness has consistently been reported by several studies [6–11], while adolescents have the desire and ability to share their values, beliefs and preferences of treatment [6, 12, 13] and parents indicate that they find it important to communicate about these themes [6]. Talking about cancer with their child has been designated as one of the most significant sources of stress during treatment [14], especially talking about what to do if the adolescent’s health should get significantly worse [11].

Advance care planning (ACP) has been widely advocated [15, 16] to support patient and family engagement in understanding the patient’s values, preferences and goals of care – regardless of prognosis and disease trajectory [17]. It entails a communication process that is aimed at aligning future medical care and treatment with an individual’s values and preferences in a timely manner, not only at the end of life but at any stage in the course of the illness [17, 18]. ACP has been positively evaluated in adults, and studies in paediatrics [18–20] show promise that it can provide an opportunity to address misconceptions, improve understanding of prognosis and prepare families for future situations [21, 22]. Moreover, knowing what is important to the adolescent can be a great relief for both parents and adolescents, leading to an increased sense of control and security [22]. Despite this, exploration of the child’s perspectives by healthcare professionals in particular appears to be difficult due to barriers such as insecurity about their own communication skills, a lack of time and perceived parental unreadiness and gatekeeping by both families and healthcare professionals [23–26].

A few paediatric ACP (pACP) programs have been developed and tested on different levels ranging from face validation to effectiveness [5, 18, 22, 27, 28]. However, these initiatives are often predominantly focused on specifying end-of-life care preferences or on providing healthcare professionals with tools, materials and training to support them in performing ACP conversations with their patients instead of the adolescents and parents themselves. This despite the fact that ACP has been widely defined as a broader approach (not limited to end-of-life topics), and that it is a process of behaviour change that is not only initiated by healthcare professionals but by other stakeholders as well [18, 29]. Thus, adolescents and their parents would likely benefit from, and increase their communication about, a broad range of topics through a structured program that empowers adolescents themselves and their parents while simultaneously involving healthcare professionals. However, such a program – including evidence of its effectiveness – does not yet exist.

The primary objectives of this study were to develop and test the acceptability and feasibility of a novel pACP intervention for adolescents with cancer and their parents. In this paper, we present both the development process and the content of the final intervention. The specific objectives of the study were:

1. To identify potential intervention components and current barriers and facilitators for ACP in the context of paediatric oncology.
2. To specify the pathway through which the pACP intervention is likely to achieve change, including the selection of proximal and distal outcomes and required intervention components.
3. To evaluate the acceptability and feasibility of the intervention components and materials with adolescents with cancer, parents and healthcare professionals.

The resulting BOOST pACP (Benefits of Obtaining Ownership Systematically Together in paediatric Advance Care Planning) intervention aims to facilitate and improve ACP communication among adolescents between 10—18 years old with any type of cancer at any stage, parents, and paediatric oncologists [30].

## Methods

### Study design and setting

**Fig. 1:**
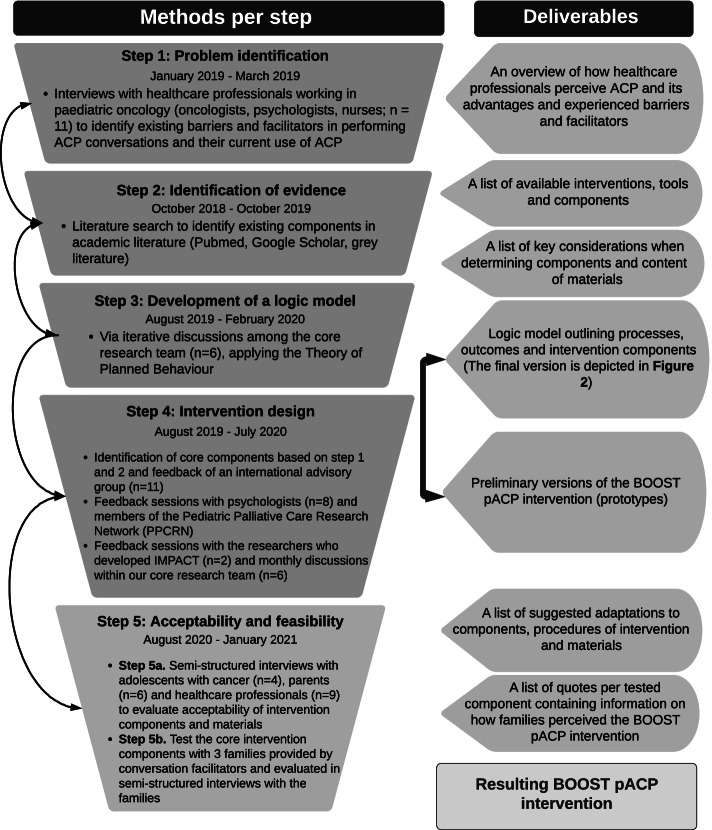
Flow diagram of the development process of the BOOST pACP intervention. The ‘core research team’ consists of: one PhD student with a background in health intervention development (AvD), two professors (PhDs) in palliative care, one sociologist (PhD), one psychologist (PhD), an assistant professor specialized in paediatric palliative care (PhD), and a paediatric oncologist (MD)

We conducted a comprehensive iterative phased approach to develop and test the BOOST pACP intervention, informed by the supplementary guidance for the development of complex interventions by Bleijenberg et al. (2018) [31]. This guidance combines elements from the development phase (phase 0 – 2) stipulated within the Medical Research Council’s (MRC) Framework for Complex Interventions [32, 33] with extra elements such as problem identification to enhance the intervention design. We applied several methods to inform the study objectives, resulting in five steps of the intervention development process (Fig. 1).

The reporting of the intervention development in this paper is compliant with the GUIDED checklist (2019)[34].

The study was performed in Flanders, the Dutch-speaking part of Belgium. In Flanders, there are four University hospitals, each with a paediatric oncology department. These paediatric departments treat children between 0 and 16 years old, and, more exceptionally, children between 16 and 18 years old. In Belgium, every year about 340 children (0–14 years) and 180 adolescents (15–19 years) are diagnosed with a malignancy [35]. In Flanders, the number of new diagnoses in the age group 10 – 18 years was 139 in 2018 (email from the Belgian Cancer Register, Brussels, 30 October 2020).

### Study participants and data collection

#### Step 1: Problem identification

We performed individual semi-structured interviews with healthcare professionals working at a paediatric oncology department in Flanders. Interviews lasted approximately 40 min and were structured according to a topic list, covering: knowledge of and experience with ACP, barriers and facilitators, use of ACP on their ward, whether any tools or materials are being used to conduct ACP, desired outcomes, and importance and feasibility of integrating ACP on the ward structurally. We first asked them what they understood ACP to be, and we used the definition of Rietjens et al. [36] to supplement their explanation. In addition, we proposed components of FACE [37] – at that point in time, the only pACP intervention that demonstrated effectiveness in paediatric oncology [5] – to get an impression of the fit of such an intervention in our local context.

#### Step 2: Identification of evidence

We performed a scoping review of academic and grey literature via PubMed and Google Scholar on existing pACP tools and interventions, barriers and facilitators in performing ACP conversations for families and healthcare professionals. The first author (AVD) performed hand searches using terms such as ‘advance care planning’, ‘goals-of-care conversations’, ‘interventions’, ‘tools’, ‘paediatrics’,’adolescents with cancer’ and selected and summarized literature.

#### Step 3 and step 4: Development of a logic model, including processes, outcomes and intervention components

The process of developing and adapting the logic model (Step 3) ran synchronously with the adaptations made to the intervention components (Step 4). Whenever we adapted the logic model, the components were adapted accordingly. Decisions about adaptations were made by reaching consensus within the core research team through monthly meetings (in total, approximately ten meetings were held). Several activities were performed chronologically to develop and refine both the logic model and, accordingly, the intervention components:

1. Identification of core components and proposing these to an international advisory group: Intervention components are defined as the parts of the complex intervention that are distinct from, but compose the whole of the intervention in full or in part [38]. We first identified five core components of the pACP intervention, based on the results of the interviews with healthcare professionals and the literature review (step 1 & 2). We contacted researchers and healthcare professionals working in the pACP field worldwide and sent them an e-mail asking them to give feedback on our identified components. We included the underlying evidence and rationales for each of the five components and gave selected members the option to give feedback in any way they preferred. Guiding questions presented to them were: “Are any important components missing?”; “Which components do you find most important?”; and “Do you acknowledge and support our findings?”. We included the feedback of this international advisory group in the following phases of the development process. Researchers who were in the process of developing a pACP intervention, or who have studied the effects of pACP, were contacted further by digital meetings. These researchers included the developers of pACP interventions IMPACT [18] and FACE [5, 39].
2. Development of the logic model: After discussions within our core research team, we developed a preliminary logic model to define desired outcomes and to be able to specify pathways through which the pACP intervention would change desired outcomes.
  We applied the Theory of Planned Behaviour (TPB) [40] as a guiding framework for the development of the logic model, integrating the results from previous steps 1 and 2. The TPB postulates that intention, the most important determinant of behaviour, is in turn determined by conceptually independent constructs such as attitude and self-efficacy [40]. The TPB was chosen because it has proven to be a useful framework for designing behaviour change interventions and for explicating the mechanisms by which interventions are expected to exert their effects on behaviour [41]. In developing and adapting the logic model, we strove to fulfil three conditions for effective behaviour change: i.e., the target concepts must be: 1) determinants of behaviour; 2) amenable to change via intervention; 3) able to be translated into a practical application in a way that preserves the parameters for effectiveness and fits with the target population, culture and context [42]. The ACP process is complex – for instance, it involves many different actors, such as the patient, informal caregivers, and different kinds of healthcare professionals who ideally engage in different ACP behaviours [43]. This indicates the need for a complex intervention including multiple components, complicated or multiple causal pathways, feedback loops and mediators or moderators of effect, and potentially targeting multiple groups of participants [44].
  The preliminary logic model and draft intervention components were proposed to: 1) psychologists purposively recruited via a special Facebook page for psychologists in Flanders, snowball sampling and by contacting the paediatric oncology departments of the hospitals. We decided to include feedback from psychologists due to the psychosocial aspects of ACP and their expertise in facilitating communication, and we met with them individually; and 2) members of the Pediatric Palliative Care Research Network (PPCRN), an interdisciplinary, multi-centre team of researchers and clinicians specialized in paediatric palliative care, set up by the Dana-Farber Cancer Institute (USA).
3. Feedback sessions with researchers experienced in paediatric ACP intervention development**:** two additional feedback sessions were held with two experienced researchers in paediatric palliative care who developed the IMPACT ACP intervention [18] in the Netherlands (MK and JF). We involved them in the BOOST pACP intervention development process from the moment we noticed that the IMPACT goal and materials represented a good fit with the logic model (see Barriers and facilitators for pACP in the context of paediatric oncology and identification of existing tools and interventions to identify components (objective 1) ).

#### Step 5: Testing of acceptability and feasibility of the intervention

Within this study, the term ‘acceptability’ refers to determining how well an intervention is received by the target population and the extent to which the new intervention, or its components, meets the needs of the target population and organizational setting [45]. To test acceptability of the intervention materials (Step 5a), we performed semi-structured individual interviews with the target groups: i.e., adolescents who are currently being treated for cancer or individuals who finished treatment in their adolescence, parents of adolescents with cancer, and healthcare professionals working at a paediatric oncology department (nurses, psychologists and oncologists). Participants were recruited via paediatric oncologists and psychologists from two University Hospitals and support organizations that posted a flyer on their social media. Interviews took place either at the participant’s home, or online (in conformance with the local COVID-19 measures). During these interviews, intervention materials were presented on paper or online to the participants for them to review. We discussed in depth the relevance and formulation of the themes and main questions mentioned on the intervention materials with the adolescents, parents and healthcare professionals. If questions were perceived as confrontational, the core research team re-evaluated the risk of formulating questions and phrases that negatively affect the relationship in the conversation, on the one hand, to the degree to which this was important to the goal of the study, on the other hand. This helped us to decide whether to adapt the formulation or remove the text entirely. In addition, two psychologists specialized in working with adolescents gave feedback on the materials.

In this study, ‘feasibility’ refers to the extent to which the intervention will be able to be delivered as intended [32]. We tested the core intervention components with three families in the feasibility test [46] (Step 5b). The families were recruited by healthcare professionals in the University Hospitals of Ghent and Brussels. AVD performed semi-structured interviews regarding their experiences.

All interviews (Steps 1, 4 and 5) were audio-recorded and transcribed. We applied thematic analysis by hand to structure the participants’ feedback. Suggested adaptations were discussed with the core research team in multiple meetings.

### Ethical approval

The methods outlined in Step 5 were approved by the Medical Ethics Committees of the Ghent and Brussels University Hospitals (B1432020000060) in Flanders, Belgium.

## Results

Throughout the development process, we further specified the content and rationale of the BOOST pACP intervention, which resulted in several prototypes through which the intervention evolved (Additional file 1). Given the iterative nature of the work performed, results are described according to the objectives of the study, rather than the chronological steps outlined in the methods section.

### Participant characteristics

During Step 1 (Problem identification), 11 healthcare professionals working at a Flemish paediatric oncology department participated (four oncologists, two psychologists and five nurses). During Step 3 (Development of a logic model) and Step 4 (Intervention design), we contacted 17 healthcare professionals and experts working in the field of pACP worldwide, of whom 11 responded (65% response rate). Feedback on the logic model was provided by eight local psychologists, members of the Pediatric Palliative Care Research Network (PPCRN) and two experienced researchers. During Step 5a (Acceptability of the intervention), four adolescents and young adults (AYAs) who were at that time being treated for cancer or finished treatment in their adolescence, six parents, and nine healthcare professionals (nurses, psychologists and oncologists) working at a Flemish paediatric oncology department (Table 1) were involved. During Step 5b (Feasibility of the intervention), two 13-year-old adolescents and one 17-year old adolescent participated together with one parent. The core research team led and supervised the development and testing throughout all steps.

**Table 1:** Characteristics of stakeholders involved in Step 5a: acceptability of the intervention

Participants	Adolescents and young adults	Parents	Healthcare professionals
**Gender**			
Male	2	1	-
Female	2	5	9
**Age**^**a**^			
**Mean (SD); range**	19.25 (2.86); 16 – 23		
< 30 years old	-	-	2
30 – 39 years old	-	1	1
40 – 49 years old	-	5	1
> 50 years old	-	-	5
**Time since diagnosis**			
< 1 year ago	1	-	-
> 3 years ago	3	-	-
**Highest education**			
Lower secondary education	-	1	-
Higher secondary education	-	2	-
Graduate	-	1	-
Bachelor	-	2	2
Master	-	-	7
**Family situation**			
Married/living together	-	5	-
Single parent/unmarried	-	1	-
**Provider type**			
Paediatric oncologist	-	-	2
Clinical remedial educationalist	-	-	1
Clinical psychologist	-	-	4
Specialist in palliative care at home	-	-	2
**Work experience within field of paediatric oncology**			
< 5 years	-	-	4
5–10 years	-	-	1
11 – 20 years	-	-	1
> 20 years	-	-	3

Values are numbers

^a^Age categories are not applicable to young adults and adolescents as they were all younger than 23

### Barriers and facilitators for pACP in the context of paediatric oncology and identification of existing tools and interventions to identify components (objective 1)

The challenges indicated by healthcare professionals are mostly system-, attitude- or parent- related factors (Table 2), many of them corresponding with findings from the literature [18, 23, 25, 47]. Findings from the interviews confirm that ACP is complex and not only dependent on healthcare professionals’ behaviour, but also on broader patterns that should be targeted within an intervention, such as the way the family has been communicating with the medical team throughout their treatment.

**Table 2:** Barriers and facilitators for pACP from the perspective of healthcare professionals working in paediatric oncology

Barriers	Facilitators
- ACP is not yet performed structurally - conducting ACP is deemed difficult - lack of time during standard consultations - insecurity about the timing of such conversations - too little training to perform ACP conversations - lack of structure in the way the medical team works hinders involving the other team members - afraid of not being able to deal with the family’s emotions - afraid of losing the parents’ trust when discussing certain themes with the patient - perceived lack of parental readiness to talk about ACP themes - because the child’s situation can change rapidly, professionals do not always see an added value regarding starting a conversation on their current or future preferences - the idea that you are only able to discuss ACP themes when the patient or parent him- or herself opens up the conversation, or that it is necessary to perform ACP in an indirect way to almost hide what they mean, is illustrated by this quote from a participant: *“it’s an art isn’t it, to try and bring it up in a way that they don’t notice you want to talk about it.”* - the idea that, in the oncological target group, ACP is less needed and is done sufficiently due to the relatively clear illness trajectory compared to other groups with complex chronic conditions	- agreement that conducting ACP conversations is important, and that it is essential to start talking about ACP themes rather early in the illness trajectory - the view of ACP as a broader process and not only with the end goal of completing an advance directive - the belief that ACP would lead to more involvement of the adolescent in their treatment, and that the family is better informed about the different potential trajectories - the belief that ACP will give the family peace of mind, as they will have discussed ACP themes and thought about different potential trajectories, making it easier to make difficult decisions when needed - consensus about the criteria the facilitator performing the BOOST pACP conversations should adhere to: have experience with talking with adolescents with cancer, have good communication skills and have sufficient time to conduct the conversations

We identified several ACP tools and interventions in the literature: My Choices [48], Voicing My Choices [22, 49], the family-centred advance care planning (FACE) intervention [50], FINK (Family Interaction Nurtures Kids) cards [51] and the Implementing Paediatric Advance Care Planning Toolkit (IMPACT) [18]. In particular, IMPACT followed a rationale that we identified as important as it matched with findings from the interviews with healthcare professionals: namely, aiming to generate an open conversation on a comprehensive set of ACP themes among clinicians, parents and the child. IMPACT consists of a set of materials for both clinicians and families to prepare, conduct and document ACP conversations and a two-day clinician training. IMPACT can be used in early phases of the illness trajectory and is primarily focused on defining shared goals of care and treatment instead of on filling out an advance directive. The pilot evaluation of IMPACT showed that participants perceived that all of the themes mentioned in the materials were appropriate for discussion with children with life-limiting conditions and their families [18], incorporating a holistic person-centred approach and stimulating the exploration of the voice of the child. These materials also seemed applicable for our specific target group. We selected several IMPACT components with agreement from the developers, such as the preparation booklets, the ACP themes from the conversation guides, the summary sheet and the training and adapted these to the specific target group of the BOOST pACP intervention.

### The BOOST logic model (objective 2)

**Fig. 2:**
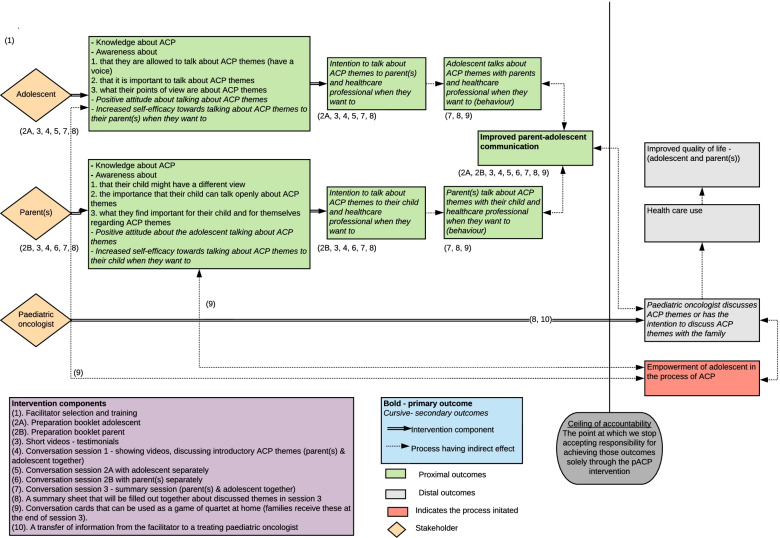
Logic model of the resulting BOOST pACP intervention. Proximal outcome = an outcome that can be realized in a short time. Distal outcome = an outcome that can be realized in the long-term [52]

The logic model of the resulting BOOST pACP intervention is displayed graphically in Fig. 2.

The main goal and primary outcome of the BOOST pACP intervention is to improve parent-adolescent communication on ACP themes. In turn, improved parent-adolescent communication on ACP themes would lead to improved communication between the family (parents and adolescent) and the paediatric oncologist, further facilitated by the transfer of information derived from the ACP conversations from facilitator to paediatric oncologist.

Following the TPB, important secondary goals of the intervention are: an increased self-efficacy towards, and a more positive attitude about, talking about ACP themes, in combination with more knowledge about ACP and awareness about their own role in the communication process. Based on this logic model, ten different intervention components were constructed.

### The resulting BOOST pACP intervention

The resulting BOOST pACP intervention comprises ten intervention components, which are summarized in Table 3 according to the TIDieR (template for intervention description and replication) checklist [53]. Table 4 gives an overview of the rationale for each of the intervention components and their specific content. The rationales are based on existing evidence and feedback from stakeholders throughout the different steps of the development process.

**Table 3:** Description of the final BOOST pACP intervention according to the TIDieR checklist

Timing	Intervention component (*n* = 10)	Supporting material(s)	Intervention activities, procedures and processes
Prior to start of program and approximately every six months	1. Facilitator selection and training	Facilitator manual outlining all steps of the conversation sessions (including extra follow-up questions for each conversation card)	- Selection of two external facilitators - Preparation of facilitators for the training - Two-and-a-half-day training program for facilitators focused on conversation skills and consisting of theoretical explanation and role plays - Debriefing sessions for facilitators (discussing challenges in case studies) approximately every six months for facilitators (four hours)
At least one week before conversation session 1	2. Preparation booklets for adolescent (2A) and parents (2B)	Separate booklet for parents and adolescent including information about ACP and questions to trigger the thinking process about ACP themes	- Data collectors give preparation booklets to the families that are assigned to the intervention group or send the booklets via e-mail - Adolescent and parent(s) have the option to read their preparation booklet before conversation session 1 takes place
During conversation session 1	3. Short videos of two families talking about their experiences with the intervention	Videos with testimonials of two families talking about their personal situation and experienced effects of the BOOST pACP intervention	- The facilitator introduces the videos that will be shown in conversation session 1 - Adolescent and parent(s) watch the videos during conversation session 1 - The facilitator asks whether the adolescent and parent(s) recognize any aspects from the videos
One – two weeks after having received the preparation booklets	4. Conversation session 1	Facilitator intervention manual and conversation cards	- The facilitator guides the conversation session and introduces the conversation cards to the adolescent and parent(s)
One – two weeks after conversation session 1	5. Conversation session 2a	Facilitator intervention manual and conversation cards	- The facilitator guides the conversation session with the adolescent alone, using the structured conversation guide outlined in the intervention manual and conversation cards. The conversation is tailored to the preferences of the adolescent (e.g., to (not) discuss ACP themes and the order)
One – two weeks after conversation session 1. Often planned on the same day as session 2a	6. Conversation session 2b	Facilitator intervention manual and conversation cards	- The facilitator guides the conversation session with the parent(s) alone using the structured conversation guide outlined in the intervention manual and conversation cards. The conversation can be tailored to the preferences of the parent(s) (e.g., to (not) discuss ACP themes and the order)
One – two weeks after conversation session 2b	7. Conversation session 3	Facilitator intervention manual	- The facilitator guides the conversation session with the adolescent and parent(s), using the structured conversation guide outlined in the intervention manual and gives room for them to discuss ACP themes
During conversation session 3	8. Summary sheet	Summary sheet on which the same themes discussed during the conversation sessions are covered. If given permission by the family, the facilitator will send the summary sheet to the paediatric oncologist to include in the patient’s electronic dossier	- The facilitator introduces and explains the summary sheet. The facilitator asks whether the family would like the information they write down to be shared with the paediatric oncologist/medical team
At the end of conversation session 3	9. Conversation cards	Conversation cards that can be used as a game of quartet at home whenever the family members would like to	- The facilitator explains the purpose of the quartet game and that the cards can facilitate communicating on ACP themes together at home in a playful way
Within one month after conversation session 3	10. Transfer of information from ACP facilitator to the paediatric oncologist involved in the care of the adolescent	The summary sheet is used during the transfer of information	- If the family has given permission, the facilitator makes an appointment with the paediatric oncologist involved in the care of the adolescent, indicated by the family - The facilitator gives a summary of the conversations with the family to the paediatric oncologist and asks whether he or she can add the summary sheet to the patient’s electronic dossier

**Table 4:** Specification of, and rationale for, the identified intervention components and materials throughout the development process

Intervention components	Rationale/evidence for the component	Illustrative quote
Facilitator	Should be external to the medical team, because the goal is to test the effectiveness of the BOOST pACP intervention in a randomised controlled trial – and otherwise there is a risk of contamination. None of the parents and adolescents we interviewed disliked the idea of discussing ACP themes with a facilitator they do not personally know yet	“We have children in our hospital that have five different treating oncologists. Which of these oncologists should be involved in the intervention and in what way? They don’t have time.” (Healthcare professional 4)
A training program for facilitators	Ongoing training of facilitators is important, both regarding conversation skills and steps necessary for the study. We added at least two debriefing sessions	“An initiation training is important, but it is also essential to provide ‘coaching on the job’ sessions.” (Researchers who developed IMPACT– respondent 1)
Multiple conversations structured by a conversation guide	ACP is a process, tailored to the needs and readiness of parents and adolescent. The conversation guide is therefore not rigid. However, it provides the necessary structure. Four conversations within three months was considered feasible by parents and adolescents. Healthcare professionals doubted whether 60 min per conversation would be sufficient	“You don’t win trust in one conversation and ACP is a process so it’s good you are proposing several conversations.” (International advisory group – respondent 3)
Patient tools (suitable for adolescents)	We added conversation cards to give structure to the conversations. Adolescents and parents liked the idea of using conversation cards and that they are able to decide themselves what themes to discuss or not to discuss. This gave them a structure but at the same time a certain freedom and opportunity to identify their own needs. Optional person-centred exercises for facilitators to use were added in case the adolescents find it difficult to respond to the questions. Conversation cards that can be used as a game of quartet were added to give the family the opportunity to continue ACP communication whenever they want to	“Conversation cards work very well to involve children in a playful way. You get different conversations when using these kinds of tools.” (Healthcare professional—respondent 11)
Documenting the outcomes of the conversations	Introducing an advance directive in the last conversation session would not fit with the goal of improving parent-adolescent communication and the step-by-step approach and broad target group of the intervention. Therefore, the content of the summary sheet matches the content of the conversation sessions	“It is very difficult for parents to put end-of-life preferences on paper in a concrete way. They associate it with giving up and think that nothing will be done to help their child anymore.” (Healthcare professional 7)
Transfer of information	The participants stressed the importance of a link between this external facilitator and the medical team to ensure continuity of care. Responsibility for transferring the information from the ACP conversations should not lie with the families themselves, but should be built into the intervention. If given permission by the family, the summary sheet is sent directly to the paediatric oncologist, and the facilitator has 30 days to plan the transfer of information with the paediatric oncologist	“If your aim is to get a result concerning ACP, you will have to make sure a healthcare professional is involved in some way in your intervention.” (Researchers who developed IMPACT– respondent 2)

Additional file 2 describes the content of the components and intervention materials in more detail.

**Fig. 3:**
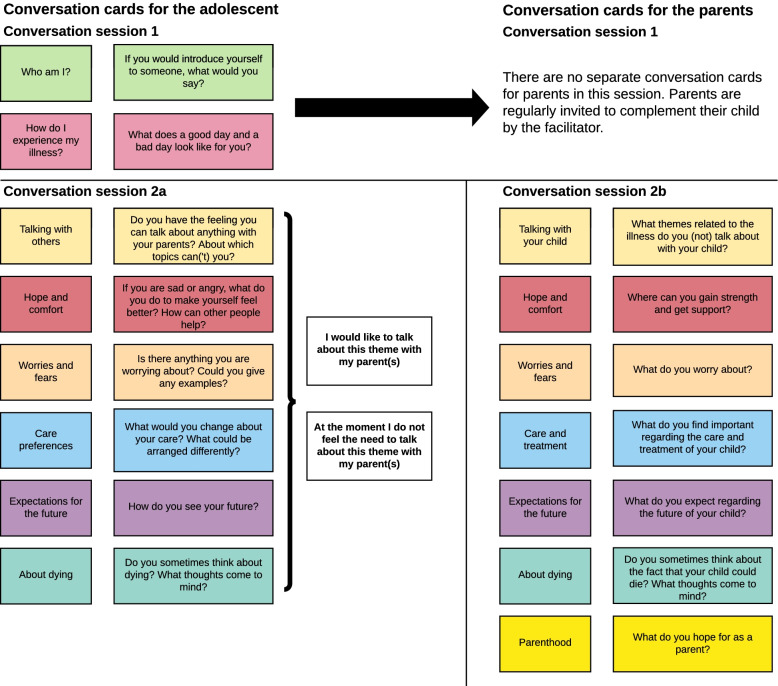
Conversation cards used by the conversation facilitators during conversation sessions 1, 2a and 2b. To integrate structure into the conversations structured by the conversation cards, one main question is printed on the back of the conversation cards. In the intervention manual, the follow-up questions are listed. Two of these follow-up questions will be asked of every participant. The remaining questions in the intervention manual can be used by the facilitator, depending on what course the conversation takes and to allow flexibility

A total of four conversation sessions are at the core of the BOOST pACP intervention: two conversation sessions are held with the adolescent with cancer and their parent(s); two of the sessions are held separately. Figure 3 depicts the conversation cards used in the conversation sessions 1, 2a and 2b.

#### Conversation session 1 (with adolescent and parent(s))

The goals of this session are to: 1) inform the family about the upcoming conversations and the BOOST pACP intervention; 2) introduce the family to the concept of advance care planning; and 3) positively affect the attitude and self-efficacy of the family regarding talking about ACP themes with one another. In this session, videos with testimonials of two families talking about their personal situation and experienced effects with the BOOST pACP intervention are shown and two introductory themes are discussed, using conversation cards.

#### Conversation session 2a (with the adolescent)

The goals of this session are to: 1) explore the adolescent’s point of view on several ACP themes and to what extent he or she has the need and opportunity to talk about these themes with his or her parents; 2) give the adolescent insight into the themes he or she would like to discuss with his or her parents and discuss ways to do this; and 3) normalize talking about ACP themes. Adolescents can choose what themes they want to discuss with the facilitator and the order in which to do so. They may choose to not discuss certain themes.

#### Conversation session 2b (with the parent(s))

The goals of this session are to: 1) explore the parents’ point of view on several ACP themes and the extent they talk about those themes with their child; 2) give the parents insight into themes they would like to discuss with their child and discuss ways to do this; and 3) normalize talking about ACP themes. Parents can choose the themes they want to discuss with the facilitator and the order in which to do so. They may choose to not discuss certain themes.

#### Conversation session 3

The goals of this session are to: 1) give the adolescent and parents the opportunity to discuss ACP themes; and 2) bring together the information from the other sessions and to fill out a summary sheet and discuss whether the facilitator may plan a transfer of information with the paediatric oncologist.

Several aspects of the intervention can be tailored according to the family’s personal preferences: 1) they can decide what ACP themes they do or do not want to discuss during the conversation sessions with the facilitator; 2) they can decide on the location of the conversation sessions: either at their home, at the hospital, or online. The last option was offered taking the persisting COVID-19 measures into account. We have developed a structure to perform the conversations in a similar way online as in real life and tested this during the feasibility test; 3) planning/timing of the conversation sessions: according to our protocol, we anticipated a maximum of two weeks between the conversation sessions. However, due to the child following treatment and experiencing side effects and parents juggling responsibilities at work, school and hospital appointments, we allow flexibility in the planning of the conversation sessions while aiming to finalize all conversation sessions within four months.

### Important adaptations for enhancing acceptability and feasibility of the BOOST pACP intervention (objective 3)

#### Adaptations to enhance acceptability: specific changes to the conversation/intervention materials 

We implemented several adaptations in the formulations of the text in the materials. For example, one of the categories was called “Parenthood” in the parents’ session. The main question formulated was “what kind of parent would you like to be?”, which was perceived as confrontational by several parents, illustrated by this quote:

“As a parent, you completely ignore your own needs. You just want to try your best to improve your child’s situation. It’s not relevant what kind of parent you would like to be. That question would be offensive to me.” [Parent 3]

To avoid immediate resistance from the parents, we decided to rewrite that question to: “what do you as a parent hope for regarding your child?”. This question elicited similar responses – namely, that they hoped their child would recover and experience few long-term effects. However, this question proved to be an appropriate opening question for this theme and was therefore retained. Similarly, regarding the questions meant for the adolescent, one healthcare professional warned to be careful regarding the question “Did you have symptoms for a long time before your diagnosis?”, because it was thought to potentially lead to feelings of guilt about why they did not go to the doctor sooner. We therefore removed that question.

In other cases, there was disagreement about whether questions were perceived as confrontational or useful. An example is: “Suppose the situation of your child worsens, what do you find important regarding his or her care?”. Due to some participants explaining that they recognized themselves in struggling with that question and therefore confirmed its relevance for the study, we retained that question. Healthcare professionals, as well as AYA’s and parents, indicated that the theme “Fears” should be changed into “Fears and worries”. Furthermore, the separate theme “Decision-making” was removed in the conversation sessions 2a and 2b, due to the participants’ explanation that they feel that they do not have many opportunities to ‘make decisions’ because they are following a treatment plan. Several questions that were still relevant were merged into the theme “Care and treatment”. For some ACP themes, the questions that were listed as follow-up questions at first proved to be more appropriate for eliciting responses compared to the main question. Therefore, in those cases, the order of the questions was changed.

#### Feasibility outcome: Families were positive about the tested BOOST pACP intervention components

In the feasibility test, the families received the preparation booklets a week before the first and only ACP conversation, including the videos and filling out the summary sheet. The facilitator performed the transfer of information with the paediatric oncologist. The three families who participated in our feasibility test reported appreciation of the components and found the materials applicable to paediatric oncology as illustrated by direct quotes (Additional file 3). Although we tested only a part of the intervention, some family members indicated that they experienced added value and thought it was a pleasant conversation. We did not make further considerable changes to the content of the materials.

## Discussion

The BOOST pACP intervention is developed for adolescents with cancer and their parents to improve parent-adolescent communication on ACP themes and to increase the adolescents’ involvement in their own care and treatment. We applied an iterative step-by-step approach during which adolescent (ex)patients, their parents, healthcare professionals and pACP experts were involved to develop and test the acceptability and feasibility of the intervention. The resulting ‘BOOST pACP’ intervention considers ACP as a broad concept to be initiated early in the illness trajectory. Using a structured format of pre-specified ACP conversation sessions, the intervention targets ACP communication behaviour between the adolescents and their parents, but involves paediatric oncologists in the process. Involving these three important stakeholders in a systematic way has not been tested in previously developed pACP interventions. The final BOOST pACP intervention consists of ten components: 1) facilitator selection and training, including a manual; 2) preparation booklets for the adolescent and parents; 3) short videos with testimonials; 4 – 7) facilitated ACP conversation sessions; 8) a summary sheet; 9) conversation cards that can be used as a game of quartet; 10) transfer of information from facilitator to the paediatric oncologist).

The BOOST pACP intervention is deemed suitable for the particular population of children and adolescents between the ages of 10 and 18 years old by the involved experts, healthcare professionals, adolescents and parents. Evidence about effects of ACP on the younger age group (10 – 13 years old) has not yet been evaluated. We specifically include this younger age group as there is evidence that they are willing to engage in conversations about care and treatment [54, 55]. Their willingness and ability to talk about their care and treatment are known to be variable though, depending on the stage of the illness and maturity of the individual child [54–56], which was also emphasized by the psychologists who were involved in the intervention design. It is a particular requirement that the facilitators who will guide those conversations should be experienced and trained in performing conversations with children of this age. In the intervention manual, we offer person-centered planning exercises [57] for younger adolescents that can be used if facilitators notice the adolescent has problems with responding to the facilitator’s questions. We do not only include adolescents with an unfavourable prognosis, as we believe that every adolescent with cancer might benefit from looking ahead to the future in light of uncertainties they might have experienced or will experience in the future [2]. That is why the BOOST pACP intervention regards ACP as a communication process not only about care and treatment preferences, but also about broader themes, such as what they find important in their life and what aspects of their illness they find most cumbersome. The intervention is set up in such a way that themes that are addressed within these conversations can be tailored to the adolescent’s individual situation and readiness.

As outlined in the logic model, the primary outcome of the BOOST pACP intervention is to improve parent-adolescent communication on ACP themes to eventually contribute to normalizing ACP conversations in the illness trajectory. This might seem unconventional because better parent-adolescent communication does not necessarily directly lead to a better match between the treatment and care and the adolescent’s preferences, and thus it has not been previously tested as a result of a pACP intervention. However, literature suggests that ACP is relational – meaning that it is enacted less as an individual directive and more as a family-centred and social process [58], especially in the paediatric setting [59]. Moreover, there is evidence that there is significant room for improvement in parent-adolescent communication about themes related to the illness, treatment and living with the illness – and this was also indicated by the adolescents and parents we interviewed. Parent–child communication concerning prognosis and goals of care specifically is associated with various positive outcomes, such as positive adaptation [14], lower withdrawn/depression scores [60], and patient and family well-being [61]. A broad array of themes influence goals of care, such as how the illness and treatment are experienced, what their fears and worries are, what helps them in coping and what their expectations for the future are [18]. These themes are represented in the BOOST pACP intervention in the form of conversation cards.

The BOOST pACP intervention was constructed following two guiding principles:

1. ACP is an ongoing communication process [6, 29, 58] rather than sporadic conversations when there is bad news. We therefore aimed to either initiate ACP communication early or build further on existing ACP communication, recognizing that, to be able to think about and talk about future care preferences, this must be discussed in relation to the present and the past [62].
2. ACP should be tailored to the needs and readiness of the adolescent as well as the parents. The conversation cards give them the stage to talk about ACP themes by triggering the thinking process. At all times, participants can indicate verbally that they want to stop. Although contemplating sensitive issues regarding the future – like hopes, fears and worries – is a demanding and sometimes burdensome endeavour for parents, it is important to consider that this parental unease does not reflect unwillingness to talk about these issues [62].

We have invested a considerable amount of time in identifying interventions and tools that have been developed elsewhere that have the possibility of being adapted to our local paediatric oncology context, as emphasized in the update of the framework for developing and evaluating complex interventions of the Medical Research Council [33]. Within this research study, we used the Theory of Planned Behaviour (TPB). In research regarding advance care planning and other themes related to end-of-life care, the use of behavioural theories has been reported to be still limited (although use is increasing) [63]. As many behaviours can determine the quality of care, the more extensive use of underlying behavioural theories may be warranted if we want to better understand and influence behaviours and normalize ACP. The development of the logic model has helped us to define desired outcomes and to specify pathways through which the BOOST pACP intervention can potentially achieve change [40]. Reviews of research on changing a variety of health behaviours have shown that interventions based on theory or theoretical constructs are more effective than those not using theory [64]. Before we tested the acceptability and feasibility of the BOOST pACP intervention with the end-users, we processed feedback on the formulation of ACP themes and questions in the intervention manual from the two psychologists specialised in working with adolescents who we hired as intervention facilitators. Their involvement supported a feeling of ownership and familiarization with the BOOST pACP intervention, which increases the chance of high motivation to perform an intervention that they support and to enhance implementation fidelity.

Our detailed overview of the development process of the BOOST pACP intervention can serve as an example for other researchers that aim to develop a complex intervention in a limited timeframe of two years. We have experienced that applying a step-by-step approach in different stages – during which the intervention is increasingly taking shape – and collecting feedback from a variety of suitable experts and the end-users is a time-efficient and appropriate approach, which allowed us to dive deeper into the core of which behaviours are targeted and likely to change. The combination with the use of a logic model facilitates the process of composing components that match predefined goals. This can increase the chance of an intervention having desired effects and improves the sustainability of those effects.

The study has several limitations. First, due to BOOST pACP being a complex intervention focusing on communication, eligibility is limited to Dutch-speaking participants – and so our study lacks cultural diversity and its generalisability is limited. In the participating hospital wards, there are many families that do not speak Dutch and are thus unable to participate in ACP supported by BOOST. Second, we experienced difficulties in recruiting adolescents with cancer within the age range of our target group. We had hoped to interview more adolescents regarding the acceptability of the components and intervention materials and we included two individuals who had cancer in their adolescence instead. The inclusion of these two individuals does not necessarily have to be a disadvantage, because they have the ability to consider what ACP themes they would have found important to discuss in hindsight and what formulations are preferred. Although we did not use participatory methods to include adolescents with cancer in the early conceptualization of our BOOST pACP intervention, we integrated knowledge and experience of development projects with a comparable target group: namely, adolescents with a life-threatening illness. We will have to research applicability and required adaptations for adolescents with other complex chronic conditions and younger and adult populations.

In the next steps, the intervention will be tested in a multi-centre parallel-group randomised controlled trial, with an embedded mixed-methods process evaluation in paediatric oncology in Belgium [30]. This will result in more knowledge of the effectiveness of improving ACP communication between adolescents, parents and paediatric oncologists and on other potentially positive or negative effects. We will gain insight into how adolescents with different types of cancer in various stages and parents value advance care planning and shape this communication process in the semi-structured BOOST pACP format, leading to a better understanding of ways to facilitate a tailored ACP approach. Furthermore, we will improve the components and materials according to the participants’ experiences and recommendations. In the future, if the BOOST pACP intervention proves to have positive effects, the task of facilitator could be carried out by a member of the medical team.

## Supplementary Information

**Additional file 1. **Evolution of specifications and adaptations to the BOOST pACP intervention components.

**Additional file 2. **Content of BOOST pACP intervention materials.

**Additional file 3. **Illustrative quotes from adolescents and parents who participated in the feasibility test (families n=3), on the BOOST pACP core components.

## Data Availability

The data are not publicly available because they are containing information that could compromise the privacy of research participants in this study. Data are available from the corresponding author on reasonable request.

## Abbreviations

ACP: Advance care planning
pACP: Paediatric advance care planning
PACS: Parent-Adolescent Communication Scale
RCT: Randomised controlled trial
MRC: Medical Research Council
TIDieR: Template for intervention description and replication
AYA: Adolescents and young adults
