# Supplementary material for: The BOOST paediatric advance care planning intervention for adolescents with cancer and their parents: development, acceptability and feasibility
Source: BMC Pediatr. 2022 Apr 15;22:210. doi: 10.1186/s12887-022-03247-9 (PMC9010242; doi:10.1186/s12887-022-03247-9)
Supplement: Supplementary file 1 — Additional file 1. Evolution of specifications and adaptations to the BOOST pACP intervention components. [file 12887_2022_3247_MOESM1_ESM.pdf]

Additional file 1. Evolution of specifications and adaptations to the BOOST pACP intervention components

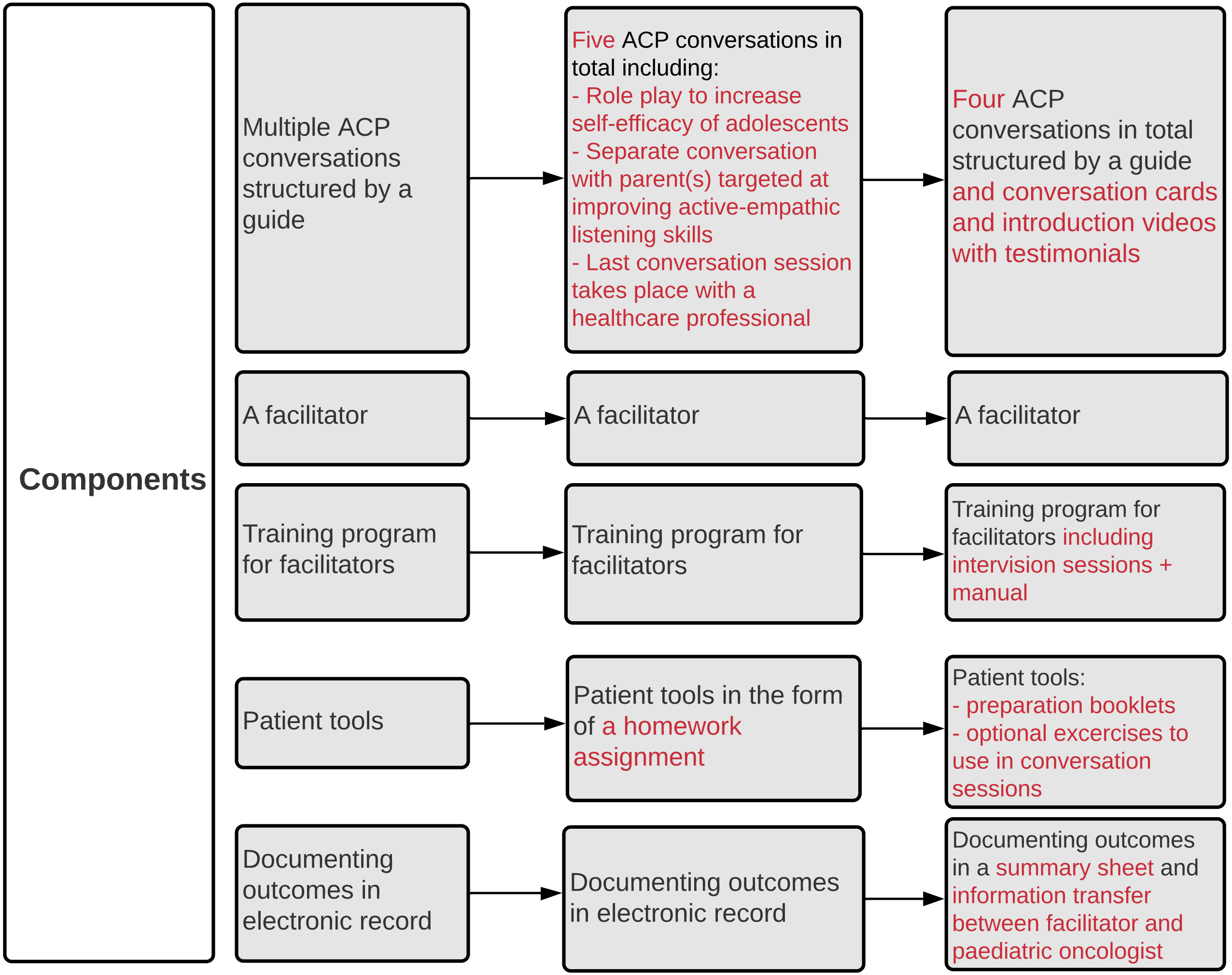

Text in red highlights the specifications/adaptations to the BOOST pACP intervention components
