# Supplementary material for: The BOOST paediatric advance care planning intervention for adolescents with cancer and their parents: development, acceptability and feasibility
Source: BMC Pediatr. 2022 Apr 15;22:210. doi: 10.1186/s12887-022-03247-9 (PMC9010242; doi:10.1186/s12887-022-03247-9)
Supplement: Supplementary file 2 — Additional file 2. Content of BOOST pACP intervention materials. [file 12887_2022_3247_MOESM2_ESM.docx]

# Additional file 2. Content of BOOST pACP intervention materials

| **Component and accompanying supporting material(s)*** | **Content of the material(s)** |
| --- | --- |
| 1. Facilitator  a) Facilitator training | Core training: Start-training program and accompanying PowerPoint presentations used for the 2.5-day training of the ACP facilitators. The training sections:  - Relevant context – 1 day:   - Introduction to ACP - The BOOST pACP intervention and its components - Family adjustment and coping strategies when facing paediatric cancer [External speaker – Marieke Van Schoors] - Paediatric advance care planning by using IMPACT [Speaker co-author JF] - Oversight of general course of events in the Flemish hospitals (local context)   - Training conversation skills [External communication experts – Wilde Kastanje Training and Education, the Netherlands] – 1.5 days  Intermediate debriefing sessions - goals:  - Exchange of experience by discussing selected fragments of conversations  - Reflecting on the goals of the intervention and on behaviour: what should I maintain, what can I do differently? How did I cope with challenges?  - Discuss in-depth what happened in the conversations and how the way facilitator’s response may have impacted these situations |
| b) Facilitator manual | A manual for trained ACP facilitators who will perform the ACP conversations with adolescents and parent(s), including a description of steps to follow during and between the structured ACP conversation sessions. The sections of the manual:  - Timeline of the study procedure for families in the intervention group  - Preparation of the conversations  - Steps to follow during the different conversation sessions  - Transfer of information  - Using person-centred exercises  - General communication tips (summary of literature and from the training)  - Extra suggestions for difficult conversations |
| 2. Preparation booklets  a) For adolescents | Adolescents can choose to fill out these booklets or only read them in preparation for the BOOST pACP conversation session.  Title: Advance Care Planning  What do you find important and how can we best take care of you?   - **About you** - What are your hobbies or interests? What do you love to do? - What is important to you? And who are important people to you? - What does your situation look like at the moment? Has your situation changed? What are you missing at the moment? What would you like in the future? - **About your illness** - What would you like to know about your illness? Do you have sufficient information? - Are there things about your illness you would like to know or would not like to know? - What do you dislike most about your treatment? - Are there any pleasant moments or experiences during your illness? - Are there things you are uncomfortable with or things you are dreading? - Do you sometimes worry about the treatment? - **About how we can help you** - How can your parents and the healthcare professionals help you? Is there anything they should take into consideration? - How can the people in the hospital help you? Is there anything they should take into consideration? |
| b) For parents | Parents can choose to fill out these booklets or only read them in preparation for the BOOST pACP conversation session.  Title: Advance Care Planning  What do you find important for your child?   - **About your child** - What makes your child happy? - What is important to your child? - What is difficult for your child? - **About your child’s illness**   - What would you like to know about your child’s illness?   - Do you want to know more about the future?   - Should the treatment not have the positive results you hoped for, what things do you want to be prepared for? - **What do you wish for your child** - What do you want for your child? - What do you worry about? - Do you want to know more about the future? - **Care and treatment** - What do you find important with regard to the care and treatment of your child? What does your child find important with regard to his/her care and treatment? - What do the healthcare professionals need to know about your child that could be important regarding the care and treatment? - If the treatment would not work well, what do you want or don’t you want for your child? |
| 3. Two short videos to be shown during the first ACP conversation session | Two families talk about their personal situation and experienced effects of the ACP intervention. Both scenarios describe that communication with each other about the illness and treatment was difficult for them and how several intervention components helped them in talking about such themes more.  Two cases:   - The first case is about a mother and her 13-year-old daughter who has been diagnosed recently - The second case is about a mother, father and their 16-year-old son who was diagnosed a few years back and has relapsed. |
| 4. Summary sheet | The summary sheet covers similar themes that are covered in the conversation sessions. The facilitator guides the family in filling out this summary sheet and the family may decide whether this is shared with the medical team. If agreed, the facilitator schedules a transfer of information with the paediatric oncologist.  We ask families to fill out their response on the summary sheet existing of the following statements:  - I am someone who…  - The illness means to me that…  - The illness means to our family that…  - As a family, we generally talk about the following themes…  - We need to keep in mind to continue talking about…  - I sometimes worry about…  - Regarding my care and treatment, it is important to me that…  - My expectations for the future are…  - If my health deteriorates, it is important to me that…  - Sometimes, these thoughts about dying come to mind…  - I would like to schedule a conversation with my oncologist:   - Yes - No, at this moment I don’t feel the need to   - Healthcare professionals are allowed to receive this information:   - Yes - No   - I would like to add that… |
| 5. Conversation cards that can be used as a quartet | Conversation cards that can be used as a game of quartet at home whenever the family wants. Families will receive these cards at the end of session 3. Each theme consists of four questions. The different themes covered by the game of quartet are:  - How do I experience the illness  - Talking to others  - Hope and comfort  - Worries and fears  - What care and treatment do you want?  - Expectations for the future  - About dying  - Do it yourself (category with different active assignments) |

*This overview excludes the conversation cards used in sessions 1, 2a and 2b. These are presented in **Figure 3** in the main paper.
