# Supplementary material for: The BOOST paediatric advance care planning intervention for adolescents with cancer and their parents: development, acceptability and feasibility
Source: BMC Pediatr. 2022 Apr 15;22:210. doi: 10.1186/s12887-022-03247-9 (PMC9010242; doi:10.1186/s12887-022-03247-9)
Supplement: Supplementary file 3 — Additional file 3. Illustrative quotes from adolescents and parents who participated in the feasibility test (families n=3), on the BOOST pACP core components. [file 12887_2022_3247_MOESM3_ESM.docx]

**Additional file 3. Illustrative quotes from adolescents and parents who participated in the feasibility test (families n=3), on the BOOST pACP core components**

|  | **Illustrative quotes** |
| --- | --- |
| **Adolescents** | |
| Videos | “I recognized myself in the videos, because I cannot talk very well about certain themes with my father. And also in the second video about the boy who decided not to have further treatment. This was also recognizable because I don’t want immunotherapy.” [17-year-old girl with acute lymphoblastic leukaemia] |
| Conversation cards and themes discussed | “In the beginning I thought it was a bit strange, using the conversation cards. I thought, you can also just ask the question. But it was not very disturbing for me.” [17-year-old girl with acute lymphoblastic leukaemia] |
|  | “I really liked being asked how we saw my future. I don’t discuss this with my doctors.” [17-year-old girl with acute lymphoblastic leukaemia] |
| About the added value of the conversation | “For children that do not talk a lot with their parents and are guarded, I think this is of added value. But I talk about anything.” [17-year-old girl with acute lymphoblastic leukaemia] |
| **Parents** | |
| Preparation booklets | “I liked the preparation booklets. They made me think about certain themes. It was a good preparation for the conversation.” [Father of 13-year-old girl with acute lymphoblastic leukaemia] |
|  | “The preparation booklet was a good tool to think about what themes I wanted to discuss and not discuss.” [Mother of 13-year-old girl with diffuse large B-cell lymphoma] |
| Videos | “It’s not like they were acting in the videos. These were real emotions, which is important I think.” [Father of 13-year-old girl with acute lymphoblastic leukaemia] |
|  | “After seeing the videos, I had this epiphany ‘maybe I should be more concerned about that’.” [Mother of 13-year-old girl with diffuse large B-cell lymphoma] |
| Conversation cards and themes discussed | “The questions on the conversations cards completely opened up the conversation, so in my opinion that was very positive.” [Mother of 13-year-old girl with diffuse large B-cell lymphoma] |
| Summary sheet | “Sometimes it was difficult for me that the paediatric oncologist was not present. In the summary sheet, it was difficult to nuance and sometimes it seemed that we were criticizing the medical team, although we didn’t mean to. However, the facilitator guided us very well.” [Mother of 13-year-old girl with diffuse large B-cell lymphoma] |
|  | “I hope the doctors already know, but we often repeat things – for example that our daughter prefers her medication in pills instead of syrup. I’m not sure if this is in her medical file or not. A lot of communication is going very well, but I have the feeling that there is also a lot of miscommunication. The more that is in the medical dossier, the better – also for the psychologists’ follow-up in the future. However, they don’t have a lot of time to read it.” [Father of 13-year-old girl with acute lymphoblastic leukaemia] |
| About the added value of the conversation | “After our conversation, I think we realized we were all on the same page. I’ve always had that feeling. But in that week, we talked about some things that we would normally not specifically discuss. Not about heavy themes, but more about how [name child] was feeling. I’m glad we participated. For example, I had the feeling that their [referring to his partner and his child] visits to the psychologist were not that useful. Maybe because I never visit them and I don’t feel the need to. But now I’ve talked about that with them and now I know that this visit is really useful for them and they really feel better. Without this conversation, I would have never asked them.” [Father of 13-year-old girl with acute lymphoblastic leukaemia] |
|  | “The most important message for me was that it is important to talk to each other about these themes and that I could indicate to my child that I’m concerned but that I’m always there to talk, and that we should not hide such things from each other.” [Mother of 13-year-old girl with diffuse large B-cell lymphoma] |
|  | “We are not really thinking about the more difficult themes yet. But talking about the easier themes has also contributed. These may not be the most essential or earth-shattering things, but it was still useful.” [Father of 13-year-old girl with acute lymphoblastic leukaemia] |
